# Clearing the Path: Redefining Hepatolithiasis Removal Through Endoscopic Hepaticogastrostomy

**DOI:** 10.7759/cureus.78775

**Published:** 2025-02-09

**Authors:** Woo Suk Kim, Tasur Seen, Krishna Gurram

**Affiliations:** 1 Internal Medicine, Icahn School of Medicine at Mount Sinai, Queens, USA; 2 Gastroenterology, Icahn School of Medicine at Mount Sinai, Queens, USA

**Keywords:** endoscopic retrograde cholangiopancreatography (ercp), endoscopy intervention, hepaticogastrostomy, hepato-biliary, hepatolithiasis

## Abstract

Hepatolithiasis is a condition characterized by the presence of bile stones in the intrahepatic bile ducts. It represents a significant therapeutic challenge owing to its association with recurrent cholangitis, biliary obstruction, and potentially life-threatening complications such as hepatic abscesses and cholangiocarcinoma. Traditional treatments include hepatectomy and percutaneous transhepatic cholangioscopic lithotomy (PTCL), both of which are effective but highly invasive, whereas endoscopic approaches often leave residual stones. We report a case of recurrent hepatolithiasis with abscesses in an 80-year-old woman who had failed to respond to conventional endoscopic approaches. Endoscopic ultrasound (EUS)-guided hepaticogastrostomy was successfully performed through the transgastric approach to allow access to intrahepatic stones for clearance with complete symptomatic relief and no further evidence of recurrence during follow-up. This case demonstrates the use of hepaticogastrostomy as a less invasive alternative to surgery while serving both diagnostic and therapeutic purposes. Further studies are needed to delineate its role as well as its long-term efficacy in the management of hepatolithiasis.

## Introduction

Hepatolithiasis represents the presence of bile stones in the intrahepatic bile ducts proximal to the confluence of the hepatic ducts. It is considered a significant cause of recurrent cholangitis and biliary obstruction [1]. This condition is predominantly found in East Asia, with incidence rates between 2% and 25% in Asian countries compared to approximately 1% in the West [2]. The disease has been associated with low socioeconomic class due to a diet mainly consisting of high carbohydrates, low protein, and low fat. However, incidence rates in East Asia have been decreasing with the improvement in nutrition and the Westernization of diet and public health policies. On the other hand, the disease is rare in the West, and it most often presents as secondary hepatolithiasis associated with underlying conditions causing strictures of the bile ducts or stasis of bile, such as primary sclerosing cholangitis, benign post-surgical strictures, malignancy, or choledochal cysts [3]. Nevertheless, incidence rates in the West have been increasing with migration and advancements in diagnostic capabilities [4].

Clinically, hepatolithiasis usually presents with the triad of right upper quadrant abdominal pain, fever, and jaundice, with further complications such as intrahepatic abscesses, biliary strictures, and increased risk for cholangiocarcinoma [5]. Diagnosis is established through a combination of laboratory and imaging findings. Laboratory tests often yield leukocytosis with neutrophil predominance and elevated liver function tests, including alkaline phosphatase and bilirubin, suggesting an obstructive pattern. Imaging studies such as ultrasound, computed tomography (CT), or magnetic resonance imaging (MRI) can show intrahepatic or extrahepatic ductal dilation with stones, focal strictures, and segmental hepatic atrophy [6].

Treatments traditionally have relied on surgical approaches, such as hepatectomy and percutaneous transhepatic cholangioscopic lithotomy (PTCL). Endoscopic intervention has been limited to the direct visualization of stones and bile drainage through stent or balloon dilation [7,8]. These methods have often resulted in residual stones that act as a nidus for recurrent infections, with an incidence rate of 29.6% even after surgery [9].

Due to the high recurrence rate despite invasive surgeries and the rising incidence rate in the West, there is an urgent need for more effective and minimally invasive alternatives for the treatment of hepatolithiasis. To overcome these limitations in the current management, we propose a novel endoscopic approach for direct removal of hepatolithiasis by hepaticogastrostomy. This provides a less invasive alternative with improved patient outcomes and reduced recurrence, further expanding the role of endoscopy in this complex condition.

## Case presentation

**Figure 1:**
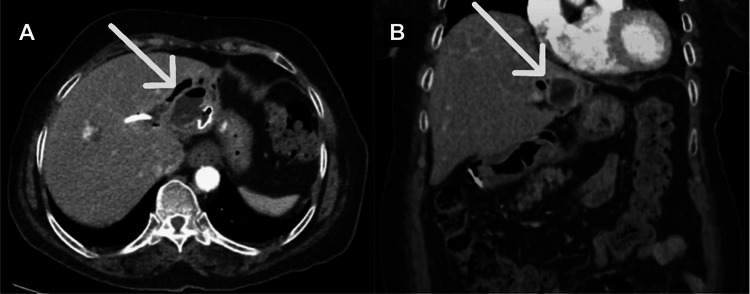
Axial (A) and coronal (B) computed tomography scans of the abdomen showing liver abscesses due to hepatolithiasis (arrows)

An 80-year-old woman was brought to the emergency department with recurrent right upper quadrant abdominal pain and fever. She had a surgical history of cholecystectomy, complicated by left intrahepatic biliary stricture and resultant hepatolithiasis, which were previously treated with endoscopic retrograde cholangiopancreatography (ERCP) with sphincterotomy and transpapillary common bile duct stent placement. However, computed tomography (CT) of the abdomen now showed multiple hepatic abscesses due to hepatolithiasis (Figure 1). The patient was treated with percutaneous drainage and antibiotics and then discharged home with a plan to repeat ERCP.

Two months later, the patient presented again with persistent fevers. A repeat ERCP confirmed a retained hepatolithiasis as the likely nidus for recurrent infections, and the intrahepatic duct stent was replaced. Despite the intervention, the patient continued to have symptoms; therefore, the decision was made to proceed with the direct endoscopic removal of the hepatolithiasis via hepaticogastrostomy.

**Figure 2:**
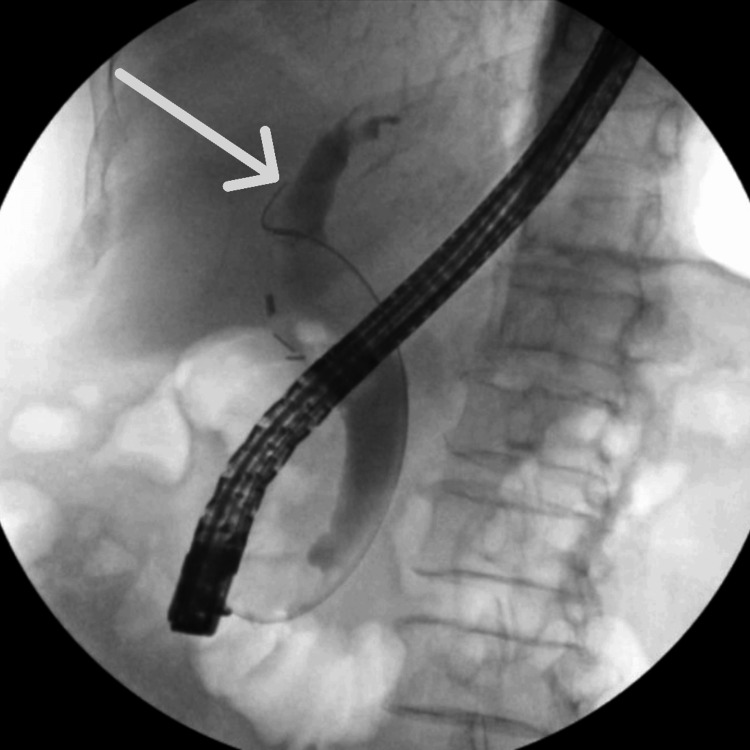
Cholangiogram showing contrast extending to the left intrahepatic bile duct (arrow)

**Figure 3:**
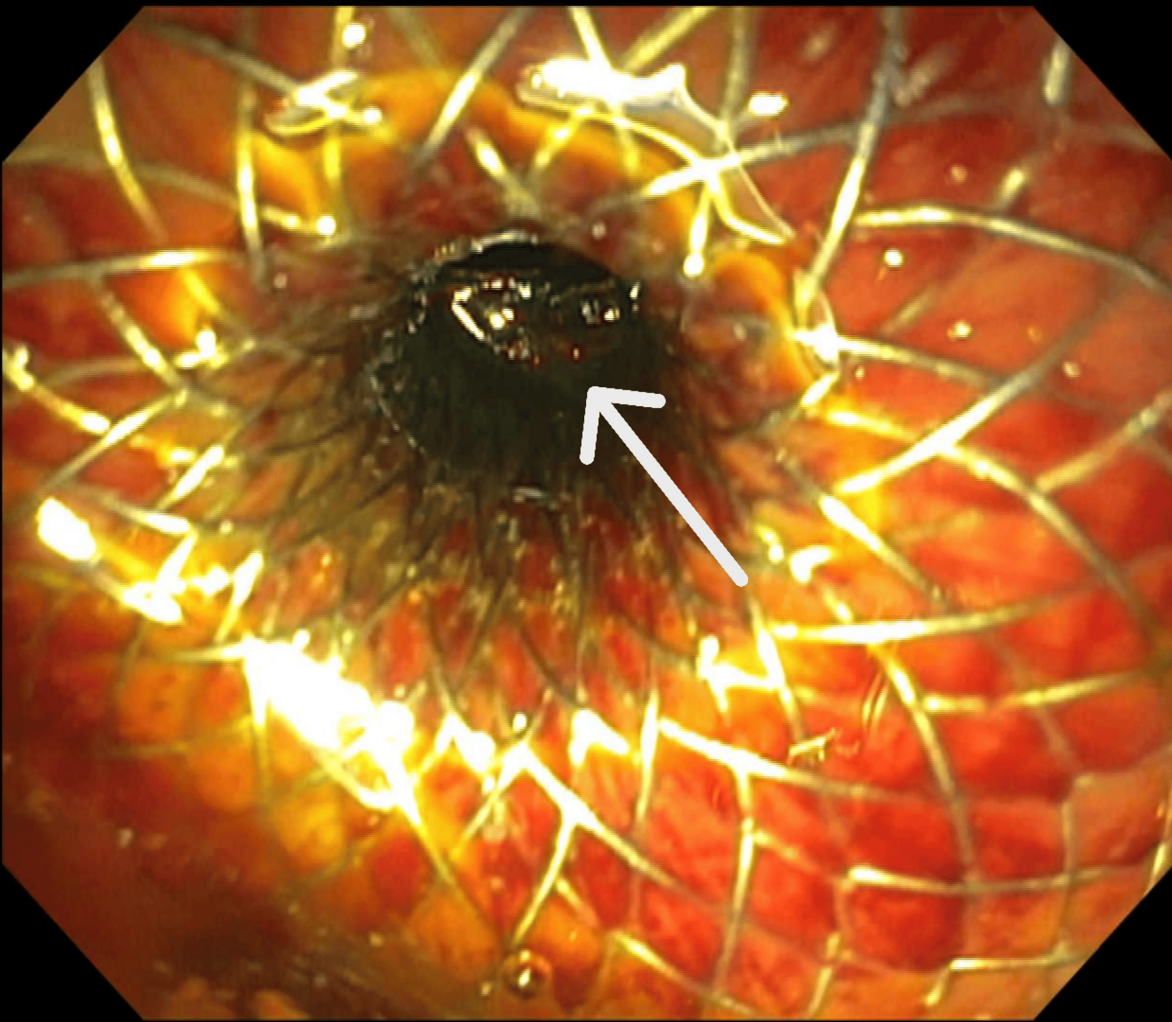
A 10 x 10 mm stent deployed in a transgastric fashion into the left intrahepatic bile duct with visualized hepatolithiasis (arrow)

The left intrahepatic bile duct was punctured using a 19-gauge needle under endosonographic guidance via a transgastric approach. The bile duct was injected with contrast, and a cholangiogram was obtained to confirm that contrast extended to the left intrahepatic bile duct (Figure 2). A 10 x 10 mm covered lumen-apposing metal stent was chosen due to the low risk of stent migration, reduced risk of leakage into the peritoneum, and large lumen diameter to ensure complete drainage of multiple hepatolithiasis [10]. It was deployed in a transgastric fashion into the left intrahepatic bile duct and dilated with a 10 mm balloon. Several stones were directly visualized and extracted by a basket (Figure 3). Due to the high stone burden, the remaining stones were left to be passively cleared through the stent. A wire was passed under direct visualization into the left intrahepatic duct, and a double pigtail stent was placed as an anchoring stent at the intrahepatic biliary ducts and the stomach. The patient returned for stent removal in two months following the successful hepaticogastrostomy with no further hepatolithiasis and resolution of her symptoms.

## Discussion

Hepatolithiasis remains a significant therapeutic challenge due to the ineffective removal of stones and its association with serious complications involving recurrent infections, hepatic abscesses, biliary strictures, and cholangiocarcinoma [1]. Although surgical approaches using hepatectomy and PTCL can offer relief in localized disease, these options are invasive and carry high risks, particularly in elderly patients with multiple comorbidities [7]. Endoscopic interventions have been largely limited to stent placement and balloon dilation, which rarely address the underlying cause and result in residual stones that act as a nidus for infection [8]. ERCP with electro-hydraulic lithotripsy (EHL) or laser lithotripsy has been demonstrated as effective, with a meta-analysis reporting an overall stone clearance rate of 88% and a recurrence rate of 13% [11]. Despite its potential, this approach has been primarily studied in patients with low stone burden and, therefore, was deferred in our patient due to the concern for extended procedural time given her multiple hepatolithiasis and comorbidities.

This case illustrates the limitation of traditional endoscopic management of hepatolithiasis and introduces a novel approach using hepaticogastrostomy for direct stone removal. Endoscopic ultrasound (EUS)-guided hepaticogastrostomy allows for precise access to the intrahepatic bile ducts with a technical success rate of 98% and a complication rate of 15-20%, including pneumoperitoneum, bilioperitoneum, infection, and stent dysfunction [12]. To minimize the risk of bile leakage, a covered stent was used in our patient. Although hepaticogastrostomy is indicated in the management of bile drainage for malignant biliary obstruction or complex benign strictures, this approach is a novel alternative for hepatolithiasis, allowing diagnostic and therapeutic intervention in a single session. Moreover, the procedural success in this elderly patient underlines the potential of this intervention as a viable alternative to invasive surgeries, especially for patients who are not optimal candidates for surgery.

## Conclusions

Hepaticogastrostomy offers a less aggressive alternative in the treatment of hepatolithiasis compared to traditional surgeries and a safer approach for high stone burden compared to more recent endoscopic advancements such as ERCP with EHL. This novel technique represents a promising advance by enabling precise localization and direct clearance of retained stones. Further studies should aim to develop standardized protocols, validate its safety and effectiveness within a greater series of cases, and define the role of hepaticogastrostomy within the overall context of hepatolithiasis management.

## References

[1] Feng X, Zheng S, Xia F, Ma K, Wang S, Bie P, Dong J (2012). Classification and management of hepatolithiasis: a high-volume, single-center's experience. Intractable Rare Dis Res.

[2] Dilek ON, Atasever A, Acar N (2020). Hepatolithiasis: clinical series, review and current management strategy. Turk J Surg.

[3] Al-Sukhni W, Gallinger S, Pratzer A (2008). Recurrent pyogenic cholangitis with hepatolithiasis-the role of surgical therapy in North America. J Gastrointest Surg.

[4] Tsui WM, Lam PW, Lee WK, Chan YK (2011). Primary hepatolithiasis, recurrent pyogenic cholangitis, and oriental cholangiohepatitis: a tale of 3 countries. Adv Anat Pathol.

[5] Sakpal SV, Babel N, Chamberlain RS (2009). Surgical management of hepatolithiasis. HPB (Oxford).

[6] Leung JW, Yu AS (1997). Hepatolithiasis and biliary parasites. Baill Clin Gastroenterol.

[7] Uchiyama K, Onishi H, Tani M, Kinoshita H, Ueno M, Yamaue H (2002). Indication and procedure for treatment of hepatolithiasis. Arch Surg.

[8] Hakuta R, Sato T, Nakai Y (2024). Balloon endoscopy-assisted endoscopic retrograde cholangiopancreatography for hepatolithiasis in patients with hepaticojejunostomy. Surg Endosc.

[9] Jan YY, Chen MF, Wang CS, Jeng LB, Hwang TL, Chen SC (1996). Surgical treatment of hepatolithiasis: long-term results. Surgery.

[10] Jain D, Patel U, Ali S, Sharma A, Shah M, Singhal S (2018). Efficacy and safety of lumen-apposing metal stent for benign gastrointestinal stricture. Ann Gastroenterol.

[11] Korrapati P, Ciolino J, Wani S (2016). The efficacy of peroral cholangioscopy for difficult bile duct stones and indeterminate strictures: a systematic review and meta-analysis. Endosc Int Open.

[12] Giovannini M (2019). EUS-guided hepaticogastrostomy. Endosc Ultrasound.

